# 

**Published:** 1892-01-30

**Authors:** 


**Notices of Births, Deaths, and Marriages**, are inserted in this column at a charge of 2s. 6d. for an announcement not exceeding four lines.

### NOTICE TO CORRESPONDENTS.

All MS., Letters, Books for Review, and other matters intended for the Editor should be addressed The Editor, The Lodge, Porchester Square, London, W.

The Editor cannot undertake to return rejected M.S., even when accompanied by stamped directed envelope.

**Notice to Advertisers and Subscribers.**

All Advertisements, Orders for Copies of The Hospital, and Business Communications should be addressed to The Publisher, *not to the Editor*, The Hospital, 140, Strand, W.C.

The Cost of Subscription, post free, is as follows:—Per week, 1½d.; per quarter, 1s. 8d.; per half-year, 3s. 3d.; per annum, 6s. 6d. Foreign:—Per annum, 8s. 8d.; payable, in all cases, in advance.

**"Burdett's Hospital Annual," 1891—1892.**

Third year of publication. 600 pp. Boards, gilt lettering. A most complete guide to British and Colonial Hospitals, Dispensaries, Nursing and Convalescent Institutions, and Asylums. Price 3s. 6d. Published by The Hospital (Limited), 140, Strand, W.C.

**NOTICE.—The Index for Vol. X. (ending September, 1891), is on sale at the Office of this Journal, price 2½d., post free.**

### GENERAL CHARITIES.

[This Column is devoted to an account of work of charitable institutions other than Medical Charities. The Editor will be glad to receive reports or news of work at 140, Strand. *Philanthropy* should be plainly written on the envelope.]

The Earl of Aberdeen will preside at the twenty-eighth anniversary dinner of the Homes for Little Boys, Farningham and Swanley, on Saturday, March 19th.

Mr. A. J. Balfour has consented to take the chair at the anniversary dinner of the Newspaper Press Fund on Saturday, May 14th, when it is expected he will be supported by a large number of stewards.

The most important offshoot of the labours of the **ST. GILES' CHRISTIAN MISSION** for the reclamation of the criminal classes is, we think, their effort to save lads from a first conviction. The importance of taking preventive measures while the character is still malleable, has become more widely recognised, and those who are experienced in the work of reclamation know full well how firmly fixed childhood's evil lessons and bent of a lifetime become unless the heart of the child can be touched before the evil has gone too far. During the past year four hundred and three boys have been admitted to the four Homes for Boys of the Mission; in addition 189 boys have been assisted, 48 have been sent to sea, 103 have been restored to their parents, and situations have been found for 562 boys. Two other important branches of the Mission are devoted to work among the lost women of St. Giles', and among discharged prisoners. Towards the work amongst the latter the Mission receives Government allowances, but notwithstanding this help the Mission is in need of funds, and donations will be gratefully received by Mr. George Hatton, 4, Ampton Street, Regent Square, W.C.

The Archbishop of Canterbury has issued a special appeal on behalf of **THE NATIONAL SOCIETY**, which has now for more than eighty years been promoting most effectively the education of the people upon the principles of the Church. The Archbishop writes: "The present time is full of warnings as to the immeasurable importance of maintaining its work and extending it. During the next few years the various Diocesan Boards of Education will be looking to the National Society to assist them in meeting the special and urgent calls which will be made upon them for the immediate improvement and strengthening of necessitous schools. The Committee have decided to open a special fund for this object, and I trust that all who regard the highest interests of the community will suffer me to commend this appeal to their earnest attention." The work of the Society is devoted to building and enlarging Church schools, to making grants towards the building and fitting of Sunday schools, to maintaining the existing Church Training Colleges, to making grants towards the salaries of Diocesan Inspectors, to diffusing the most recent and trustworthy information on educational topics, and generally to the adoption of every legitimate means for the maintenance and extension of the voluntary system of Church education. Donations will be gratefully acknowledged by the Treasurer, National Society's Office, Sanctuary, Westminster.

### SCRAPS AND GLEANINGS.

DR. VAUDREY has been elected physician to the Derbyshire Royal Infirmary.

RICHMOND and Chiswick are both in difficulties as to the provision of sites for isolation hospitals.

THE Company of Gunmakers have voted a contribution of £5 to Mrs. Hilton's Crèche and branches in Stepney Causeway.

LADY CONSTANCE HOWARD states, after careful testing, that Swinborne's isinglas is superior to any which she has ever used.

THE rental of farms owned by one of the chief London hospitals is now quoted as but £26,000 per annum, against £40,000 received a dozen years ago.

AT the annual meeting of the Eastern Dispensary a satisfactory report was given of the financial position and general working of the institution.

THE first monthly meeting of the Church of England Burial, Funeral, and Mourning Reform Association was held last week, at the Church House, Westminster.

MR. DAVID COBB, who died at Dundee last week, has left £70,000 to be applied to benevolent and charitable institutions. The Trustees have sole discretion as to the disposition of the money.

THE Edinburgh Dispensary has had a hard time this year, having been sorely taxed by influenza. The Secretary of the Samaritan Branch tells a piteous tale of destitution and want in the City.

LORD GEORGE HAMILTON, First Lord of the Admiralty, has accepted the office of trustee to the St. Andrew's Waterside Church Mission, which was held for some years by the late Mr. W. H. Smith.

THE seventeenth annual supper to destitute children, provided under the auspices of Dr. Barnardo's Homes, was given lately at the Edinburgh Castle, Limehouse, the little guests numbering nearly 3,000.

THE patients of the Brompton Hospital on Tuesday evening last enjoyed a most delightful concert through the kindness of Madame Dukas, who herself contributed two songs, and took part in a trio.

SOME of the papers warn the public against a woman who has been begging at many houses in the West-end. She appeals for clothes, which she states are necessary in order to obtain admission into the Brompton Cancer Hospital.

THE eastern wing of Salisbury Infirmary has undergone extensive alterations. Several important additions have been made, all tending to promote increased efficiency in the accommodation and greater comfort to the patients and nurses.

THE Albert Dock branch of the Seamen's Hospital, Greenwich, has lately received the munificent sum of £216 1s. 6d., the result of two variety concerts given in the Albert Music Hall in December last, and of various subscriptions, ranging from £5 to 2d.

THE sum of £264 10s. has been subscribed towards furnishing the North-Eastern Counties Friendly Societies' new Convalescent Home at Grange-over-Sands. It is very gratifying to note that the greater portion, viz., £203 18s. has been raised by friendly society members.

MR. ROBERT FREWER has received from the Treasurer of the Hospital Saturday Fund a handsome timepiece bearing the following inscription: "Presented to Robert Frewer by H. N. Hamilton-Hoare, on his retirement from the Secretaryship of the Hospital Saturday Fund. January, 1892."

THE *fête* organised by the proprietors of the *Gentlewoman*, in connection with their children's salon, and with the view of founding a cot in the Victoria Hospital for Children at Chelsea, was a gigantic success. Dog Jack attended, in his usual capacity, as collector to the Victoria Hospital.

AT the ordinary meeting of the Committee of the Seamen's Hospital (late "Dreadnought"), held on the 22nd inst., a vote of condolence with the Prince and Princess of Wales in their sad bereavement was unanimously passed. H.R.H. the Prince of Wales is a vice-patron of this national charity.

AT the memorial service to the late Duke of Clarence and Avondale, on the 20th inst., at the Parish Church, Kensington, about £37 was collected, which, after paying the incidental expenses of the service, will be equally divided among the vicars of the three poorest parishes in North Kensington, for the relief of the prevailing distress.

THE Mayor of Leicester proposes the formation of a children's league to collect subscriptions for the Children's Hospital. If twelve children collected annually twelve pennies per week, the sum total (£31 4s.) would cover the cost of one bed continuously occupied during the year. The scheme has been well received, and promises to be well supported.

A MEETING of the Executive Committee of the Waifs and Strays' Society was held yesterday, at the Church House, Westminster. General Lowry, C.B., in the chair. The Secretary reported that the Bishop of Norwich, the Bishop Designate of Carlisle, and the Suffragan Bishop of Southwark had become Vice-Presidents. The sum of £1,080 was voted for the maintenance of homes and boarded-out children.

AN animated debate took place in the Nottingham Town Council on a motion for opening the Castle Museum and Gallery on Sundays. In support of the proposal, the experience of Birmingham and other large towns was quoted. Religious bodies in the borough presented numerous petitions in strong opposition, and the resolution was rejected by 41 votes to seven, nine members remaining neutral.

THE Rev. W. D. Dalrymple, a Presbyterian missionary, has recently died at Rampur Beauleah, in Bengal, from leprosy contracted about two years ago while attending lepers. Like Father Damien, he had devoted himself to relieving the sufferings of the wretched victims of that loathsome disease, and within six months of his first coming into contact with them he perceived the signs of the malady in himself.

THE Distribution Committee of the Hospital Saturday Fund have under consideration a scheme for sending children of subscribers to the fund recovering from illness to convalescent and other homes upon a system of payment, the details of which plan will be laid before the Council at the first meeting of this year. The Lord Mayor (Alderman Evans) has consented to act as President of the fund during his term of office.

### THE STUDENT OF MEDICINE.

[All communications for this Supplement should be addressed to THE EDITOR of THE HOSPITAL, at 140, Strand, with the words, "Students' Column," written in the left hand top corner of the envelope.]

### APPOINTMENTS.

Cecil F. Beadles, M.R.C.S., L.R.C.P., appointed *pro tem.* Assistant Medical Officer to Colney Hatch Lunatic Asylum, vice G. Shaw on sick leave. J. B. Byles, L.R.C.P.Lond., M.R.C.S., appointed Junior House Physician to the Westminster Hospital. E. T. Collins, F.R.C.S., L.R.C.P., L.S.A., appointed Ophthalmic Surgeon to the Belgrave Hospital for Children. Burdon Cox, M.B., B.S., appointed House Surgeon to the Sunderland Infirmary, vice G. B. Morgan, jun., M.B., B.S. E. L. Fox, M.A., M.D.Camb., M.R.C.P.Lond., appointed Physician to the Plymouth Public Dispensary. J. Reginald Fuller, M.R.C.S., L.R.C.P., L.S.A., appointed Assistant Medical Officer to the London County Asylum, Hanwell. R. J. Hamilton, M.R.C.S., appointed Additional Honorary Surgeon to the Stanley Hospital, Liverpool. H. H. Mills, L.R.C.P.Lond., M.R.C.S., appointed Junior House Surgeon to the Westminster Hospital. George Pernet, M.R.C.S., L.R.C.P.Lond., appointed Assistant to Out-patients', Surgeon, and Physician, University College Hospital. N. H. Runciman, L.R.C.P., L.R.C.S.Edin., L.F.P.S.Glas., appointed Medical Officer for the Cork Dispensary, vice C. A. Harvey, B.A., Q.U.I., M.D. J. Simcock, M.B., B.Ch.Vict., appointed Resident Medical Officer to St. Mary's Hospital, Manchester, and the Manchester and Salford Lying-in Institution. William Stoker, F.R.C.S., M.R.C.P.Irel., appointed Surgeon to the Cork Street Fever Hospital, Dublin, vice Dr. Wharton, deceased. W. H. A. Tebbs, L.R.C.P.Lond., M.R.C.S., appointed Senior House Surgeon to the Westminster Hospital. E. Vaudrey, M.D., C.M.Edin., M.R.C.S., appointed Physician to the Derbyshire Royal Infirmary, vice Dr. Ogle, resigned. Frank A. Wagstaff, M.R.C.S.Eng., L.R.C.P.Lond., appointed Surgical Registrar to Children's Hospital, Great Ormond Street.

### VACANCIES.

The following vacancies are announced this week, the amount of salary in each case being given after the name of the institution:

### Resident Medical Officers.

Borough Lunatic Asylum, Portsmouth, Clinical Assistant for six months—Board, &c.

Bristol General Hospital, Surgeon Dentist.

Children's Hospital, Birmingham, Assistant Medical Officer—Salary, £40 per annum, with board, &c.

Coppice Asylum, Nottingham, Assistant Medical Officer, unmarried, and not more than [illegible]8 years of age—Salary £120 per annum, with board, &c.

Dental Hospital of London, Medical Tutor.

East London Hospital for Children, Shadwell, E., Medical Officer, unmarried, appointment for two years—Salary £80 per annum, with board, &c.

East London Hospital for Children, Shadwell, E., House Physician—Board, &c.

General Hospital, Birmingham, Three Assistant House Surgeons—Board, &c.

Kent County Lunatic Asylum, Chartham, near Canterbury, Medical Superintendent—Salary £600 per annum, house, &c.

London Throat Hospital, 204, Great Portland St., W., House Surgeon.

Radcliffe Infirmary, Oxford, House Surgeon—Salary £80, with board, &c, tenable for two years. Candidates must have both a medical and surgical qualification under the medical act.

### Non-Resident Medical Officers.

Bournemouth Friendly Societies' Medical Association, Assistant Medical Officer—Commencing salary £100 per annum.
General Hospital, Birmingham, Assistant Surgeon—Honorarium £100 per annum,
London Fever Hospital, Liverpool Road, Islington, N., Physician.
London Fever Hospital, Liverpool Road, Islington, N., Assistant Physician.

### ATHLETICS.

### Football.—RUGBY.

UNITED HOSPITALS v. SOUTH MIDLANDS.—Played on the 21st, at Richmond. The United Hospitals were defeated by 2 goals (10 points) to 1 goal (from a mark) and 2 tries (8 points).

PLAYED SATURDAY, JANUARY 25th.

THORNTON HEATH and CHARING CROSS HOSPITAL, at Thornton Heath drawn (1 t.—1 t.)

UNIVERSITY HOSPITAL beat WASPS (2 t.—1 t.

UPPER CLAPTON beat LONDON HOSPITAL, at Manor Park (8 g. 2 t.—0).

WESTMINSTER HOSPITAL beat 1ST SURREY RIFLES, at Camberwell (4 g. 1 t.—0).

ASSOCIATION GAME.—PLAYED SATURDAY, JANUARY 25th.

CLAPTON beat ST. BARTHOLOMEW'S HOSPITAL, at Leyton (2—1).,
MIDDLESEX HOSPITAL and KING'S COLLEGE HOSPITAL, at Wormwood Scrubs (drawn).

